# Genetic studies of fall armyworm indicate a new introduction into Africa and identify limits to its migratory behavior

**DOI:** 10.1038/s41598-022-05781-z

**Published:** 2022-02-04

**Authors:** Rodney N. Nagoshi, Georg Goergen, Djima Koffi, Komi Agboka, Anani Kossi Mawuko Adjevi, Hannalene Du Plessis, Johnnie Van den Berg, Ghislain T. Tepa-Yotto, Jeannette K. Winsou, Robert L. Meagher, Thierry Brévault

**Affiliations:** 1 Center for Medical, Agricultural and Veterinary Entomology, United States Department of Agriculture-Agricultural Research Service, Gainesville, FL USA; 2 International Institute of Tropical Agriculture (IITA), Cotonou, Benin; 3 West African Center for Applied Researches and Innovations, Lomé, Togo; 4 Ecole Supérieure d’Agronomie, Université de Lomé, Lomé, Togo; 5 Unit for Environmental Sciences and Management, IPM Program, North-West University, Potchefstroom, 2520 South Africa; 6 Biorisk Management Facility (BIMAF), International Institute of Tropical Agriculture (IITA-Benin), Cotonou, Benin; 7 Ecole de Gestion et de Production Végétale et Semencière (EGPVS), Université Nationale d’Agriculture (UNA), Kétou, Benin; 8 Faculty of Biosciences (BIOVIT), Norwegian University of Life Sciences, Ås, Norway; 9 Department for Invertebrate Pests and Weeds in Forestry, Horticulture and Agriculture, Norwegian Institute of Bioeconomy Research (NIBIO), Ås, Norway; 10 CIRAD, UPR AIDA, Centre de Recherche ISRA-IRD, Dakar, Senegal; 11 AIDA, Univ Montpellier, CIRAD, Montpellier, France

**Keywords:** Agroecology, Genetic markers, Haplotypes, Population genetics, Invasive species, Population dynamics

## Abstract

The fall armyworm, *Spodoptera frugiperda* (J.E. Smith) is native to the Americas and a major pest of corn and several other crops of economic importance. The species has characteristics that make it of particular concern as an invasive pest, including broad host range, long-distance migration behavior, and a propensity for field-evolved pesticide resistance. The discovery of fall armyworm in western Africa in 2016 was followed by what was apparently a remarkably rapid spread throughout sub-Saharan Africa by 2018, causing economic damage estimated in the tens of billions USD and threatening the food security of the continent. Understanding the history of the fall armyworm invasion of Africa and the genetic composition of the African populations is critical to assessing the risk posed to different crop types, the development of effective mitigation strategies, and to make Africa less vulnerable to future invasions of migratory moth pests. This paper tested and expanded on previous studies by combining data from 22 sub-Saharan nations during the period from 2016 to 2019. The results support initial descriptions of the fall armyworm invasion, including the near absence of the strain that prefers rice, millet, and pasture grasses, while providing additional evidence that the magnitude and extent of FAW natural migration on the continent is more limited than expected. The results also show that a second entry of fall armyworm likely occurred in western Africa from a source different than that of the original introduction. These findings indicate that western Africa continues to be at high risk of future introductions of FAW, which could complicate mitigation efforts.

## Introduction

The noctuid moth *Spodoptera frugiperda* (J. E. Smith) (Lepidoptera: Noctuidae), commonly called fall armyworm (FAW), is native to the Western Hemisphere. It is one of the principal insect pests of corn in the southeastern United States, the Caribbean, and South America and is responsible for significant economic damage in several other important crops^1^. FAW was detected in western Africa in 2016, which was followed in rapid succession by reports of infestations in most sub-Saharan nations in the subsequent 2 years^2–5^.

Perhaps the most broadly assumed explanation for this pattern of detections is that of a single introduction of FAW into western Africa in 2016, followed by rapid migration into southern and eastern Africa^6,7^. This presumed movement of large populations across thousands of kilometers and multiple natural barriers over a short period of time is considered plausible given the behavior of FAW in North America, where FAW undergoes annual migrations from overwintering sites in southern Florida and Texas to infestations as far north as southern Canada, a migratory range approximating 2500 km traversed over 1–3 months^8,9^. However, it is important to recognize that this migration occurs over multiple generations in a stepwise fashion, as FAW is a nocturnal flier and so, to the best of our knowledge, is limited to no more than 12 h of sustained flight^10,11^. These assumptions are consistent with recent laboratory studies using flight mills that showed median flight durations of between 8 and 10 h and median flight distances ranging from 21 to 38 km with a maximum of 70 km per day^12^. Modeling studies further demonstrate that the North American migration behavior is dependent on favorable seasonal wind systems that promote northward migration and agricultural patterns that provide high acreages of preferred host plants along the migratory pathway^10,11^. Whether and to what extent such conditions exist in Africa has not been described.

Alternative explanations for the appearance of rapid dissemination include multiple introductions from the Western Hemisphere or the possibility that the species has long been endemic to Africa but only recently detected. The first scenario reduces the requirement for extensive trans-continental migration while the second assumes that enhanced surveillance motivated by the initial discovery of FAW in Africa in 2016 led to its subsequent detection across the rest of the continent. Distinguishing between these explanations is important to assessments of risk and projections of economic damage. Single or multiple recent introductions represent worst case scenarios where the invasion of a new pest means there is potential for increased economic impact. Alternatively, if FAW has long been present in Africa, then its “discovery” only provides a new explanation for existing damage.

Our genetic characterizations of the African FAW populations to date are most simply explained by a single introduction into western Africa followed by rapid dispersion into most of the rest of the continent. This conclusion stems from the low genetic variation observed that suggests a recent introduction of a small invasive propagule, and the similarity in the haplotypes found at all locations that is consistent with a common source population^13–16^. In these studies, genetic variation was compared using segments from two genes, the mitochondrial *Cytochrome oxidase subunit I* gene (*COI*) and the Z-chromosome-linked gene encoding for the housekeeping enzyme *Triosephosphate isomerase* (*Tpi*). These genetic elements are particularly useful because they carry polymorphisms that are diagnostic for two major FAW groups, historically identified as strains, that are morphologically indistinguishable but differ in plant host preference and are, therefore, important considerations when assessing what crops are at risk of FAW infestations^17–19^. The C-strain (also call corn-strain) is preferentially found in corn and sorghum, while the R-strain (or rice-strain) predominates in pastures, turf grasses, millet, alfalfa, and rice^19–23^. Single nucleotide polymorphisms (SNPs) in the *COI* and *Tpi* genes are typically used to differentiate the two strains in Western Hemisphere populations, with the two markers generally in agreement^18,24,25^.

An unexpected finding from the initial genetic studies of African FAW collections was evidence of population structure, with statistically significant differences observed in both *COI* and *Tpi* haplotype frequencies between FAW populations in the western African nations of Togo and São Tomé & Príncipe compared to those from central and eastern Africa^16,26^. These results have two important implications. The first is that persistent geographic differences in haplotype frequencies in collections from similar corn-dominated habitats suggest limitations in natural migration that prevent homogenization of the African FAW populations. This is inconsistent with the hypothesis that natural migration is sufficient to explain the rapid trans-continental dispersion of FAW in Africa. The second implication has to do with the identification of the two strains. The observed differences in the *COI* and *Tpi* haplotype frequencies mean that these two markers show substantial disagreement in their determination of strain identity. As a result, it appears that *COI* is not an accurate marker of strains in Africa and that a substantial portion of the FAW population there could be derived from interstrain hybridization^14,16,26^. This is of potential significance to efforts to control FAW as the behavior of FAW interstrain hybrids has not been extensively characterized.

Understanding the history of the FAW invasion of Africa and how the genetic composition of the African FAW populations changes as it becomes established and adapts to the continent is relevant to developing strategies to limit the introduction of new invasive pests, assess the current threat posed by FAW, and to mitigate the future impact of FAW on African agriculture. To further this effort, the current paper tested and expanded on previous studies by examining collections from additional regions in Africa that included later (2018–2019) time periods. Specimens from a total of 22 sub-Saharan nations comprised of 34 collections from 2016 to 2019 were analyzed for genetic variations at *COI* and/or *Tpi* to test the consistency and stability of the previously described haplotype profiles. The results provide new insight into the magnitude and extent of FAW natural migration on the continent as well as demonstrating that western Africa continues to be at high risk of foreign introductions of FAW. This is of importance to food security in Africa as FAW subpopulations with different host preferences and resistance traits are common in the Western Hemisphere but are not currently present in Africa. Their introduction into the Eastern Hemisphere could significantly increase the risk and economic impact of FAW infestations.

## Materials and methods

**Figure 1:**
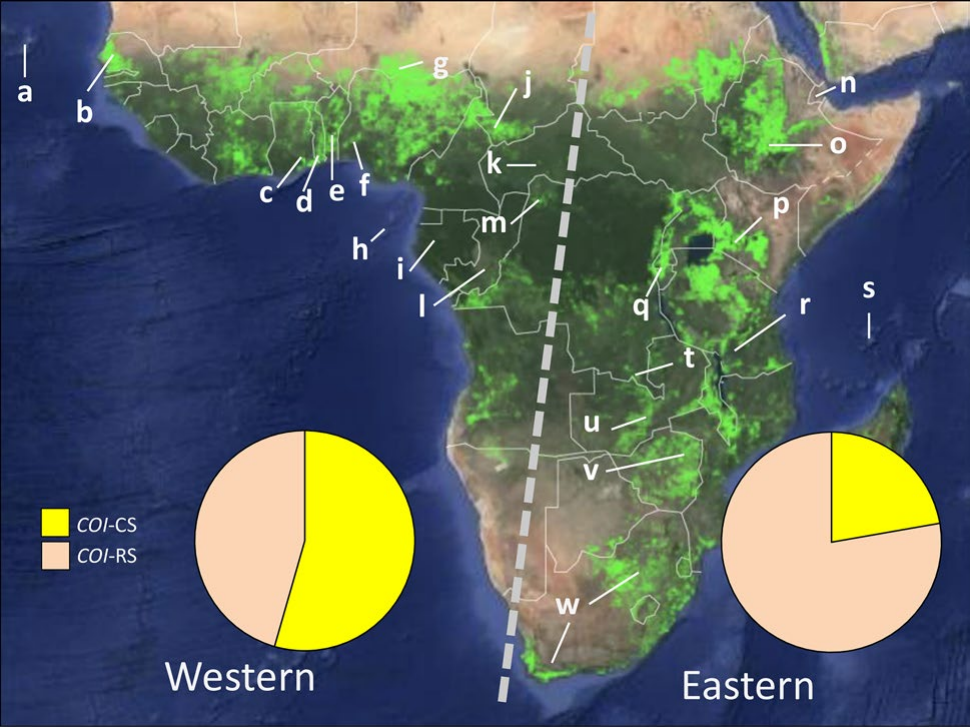
Map of croplands in sub-Saharan Africa with locations of collection sites that are detailed in Table 1. In light green are the locations of agricultural crop areas identified by satellite imagery at 30-m resolution, with the map obtained from https://croplands.org/app/map^30,31^. Dashed grey line separate western and eastern collection sites. Pie charts describe the proportion of *COI*-CS and *COI*-RS haplotypes in each region.

FAW was collected from multiple sites from 2016 to 2019, representing a total of 22 African nations (Fig. 1). Collections from 2016 to 2017 from 11 nations were previously described, with additional collections from 2018 to 2019 analyzed for four of these nations (Table 1). All specimens from Africa were larvae collected from corn except for the 2017–2018 Togo and 2018 Ghana collections that included specimens from pheromone traps. Collections from the Western Hemisphere are a combination of larval and pheromone trap collections. Both larvae and adult males from pheromone traps were stored refrigerated or air dried at ambient temperature until transport by mail to CMAVE, Gainesville, FL USA for DNA preparation.

**Table 1:** Source of FAW specimens (S&P: São Tomé & Príncipe, CAR: Central African Republic, DRC: Democratic Republic of the Congo, RoD: Republic of Djibouti).

Symbol	Year	Nation	Nearest city	Collector/[references]
a	2017	Cabo Verde	Santo Antão, Santiago	C. Tavares
b	2018	Senegal	Dakar	T. Brevault
b	2019	Senegal	Dakar	T. Brevault
c	2016	Ghana	Multiple locations	^26^
c	2017	Ghana	Multiple locations	G. Goergen
c	2018	Ghana	Ejura	G. Tepa-Yotto^27^
d	2016	Togo	Multiple locations	^28^
d	2017	Togo	Lomé	^29^
d	2018	Togo	Vogan	^27^
d	2019	Togo	Kovie	^27^
e	2017	Benin	Setto	G. Goergen
e	2018	Benin	Hougbo	G. Goergen
e	2019	Benin	Bohicon	G. Goergen
f	2016	Nigeria	Ibadan	G. Goergen
g	2019	Niger	Djiratawa	G. Tepa-Yotto
h	2016	S&P	Pinheira	^16^
i	2018	Gabon	Multiple locations	D. K. Mouendou
j	2017	Chad	Bébédjia	^26^
k	2017	CAR	Sekia	^26^
l	2018	Congo	Apoko	Louhouari & Mapangou
m	2017	DRC-North	Gemena	^16^
n	2017	RoD	Holhol	G. Goergen
o	2017	Ethiopia	Awash Melkasa	G. Goergen
p	2017	Kenya	Multiple locations	^16^
q	2017	Burundi	Multiple locations	^16^
r	2017	Tanzania	Morogoro, Songea	^16^
r	2019	Tanzania	Morogoro	J. Van den Berg
s	2018	Comoros	Mohéli	G. Goergen
t	2017	DRC-South	Kambove	^16^
u	2017	Zambia	Serenje	^26^
v	2017	Zimbabwe	Harare	G. Goergen
w	2017	South Africa	Multiple locations	^26^
w	2018	South Africa	Malelane	J. van den Berg
w	2019	South Africa	East London	H. du Plessis
Brz	2008	Brazil	Multiple sites	^26^
PR	2009–2012	Puerto Rico	Multiple sites	^26^
TX	2008–2015	Texas, USA	Multiple sites	^26^
FL	2008–2015	Florida, USA	Multiple sites	^26^

### DNA preparation and PCR amplification

DNA from individual specimens were isolated as previously described^27^. In brief, specimens were homogenized in 1.5 ml of phosphate buffered saline (PBS, 20 mM sodium phosphate, 150 mM NaCl, pH 8.0) using a tissue homogenizer (PRO Scientific Inc., Oxford, CT) or hand-held Dounce homogenizer then pelleted by centrifugation at 6000*g* for 5 min. at room temperature. The pellet was resuspended in 800 µl Genomic Lysis buffer (Zymo Research, Orange, CA) and incubated at 55 °C for 15 min, followed by centrifugation at 10,000 rpm for 5 min. Nucleic acids were purified from the supernatant using spin-column chromatography according to manufacturer’s instructions (Zymo Research, Orange, CA).

Polymerase chain reaction (PCR) amplification was performed using a 30-µl reaction mix containing 3 µl of 10× manufacturer’s reaction buffer, 1 µl 10 mM dNTP, 0.5 µl 20-µM primer mix, 1 µl DNA template (between 0.05 and 0.5 µg), 0.5 units Taq DNA polymerase (New England Biolabs, Beverly, MA) with the remaining volume water. The thermocycling program was 94 °C (1 min), followed by 33 cycles of 92 °C (30 s), 56 °C (45 s), 72 °C (45 s), and a final segment of 72 °C for 3 min. Amplification of *CO1* used the primer pair *CO1-891F* (5′-TACACGAGCATATTTTACATC-3′) and *CO1-1472R* (5′-GCTGGTGGTAAATTTTGATATC-3′) to produce a 603-bp fragment. Amplification of the *Tpi* region was done with the primers *Tpi412F* (5′-CCGGACTGAAGGTTATCGCTTG-3′) and *Tpi1140R* (5′-GCGGAAGCATTCGCTGACAACC-3′) that spans a variable length intron to produce a fragment with a mean length of 500 bp. Primers were synthesized by Integrated DNA Technologies (Coralville, IA). Gel electrophoresis and fragment isolation were done as previously described ^24^. DNA sequencing was performed directly from the gel purified PCR fragments by Sanger sequencing, using primers *CO1-924F* or *Tpi412F* (Genewiz, South Plainfield, NJ).

**Figure 2:**
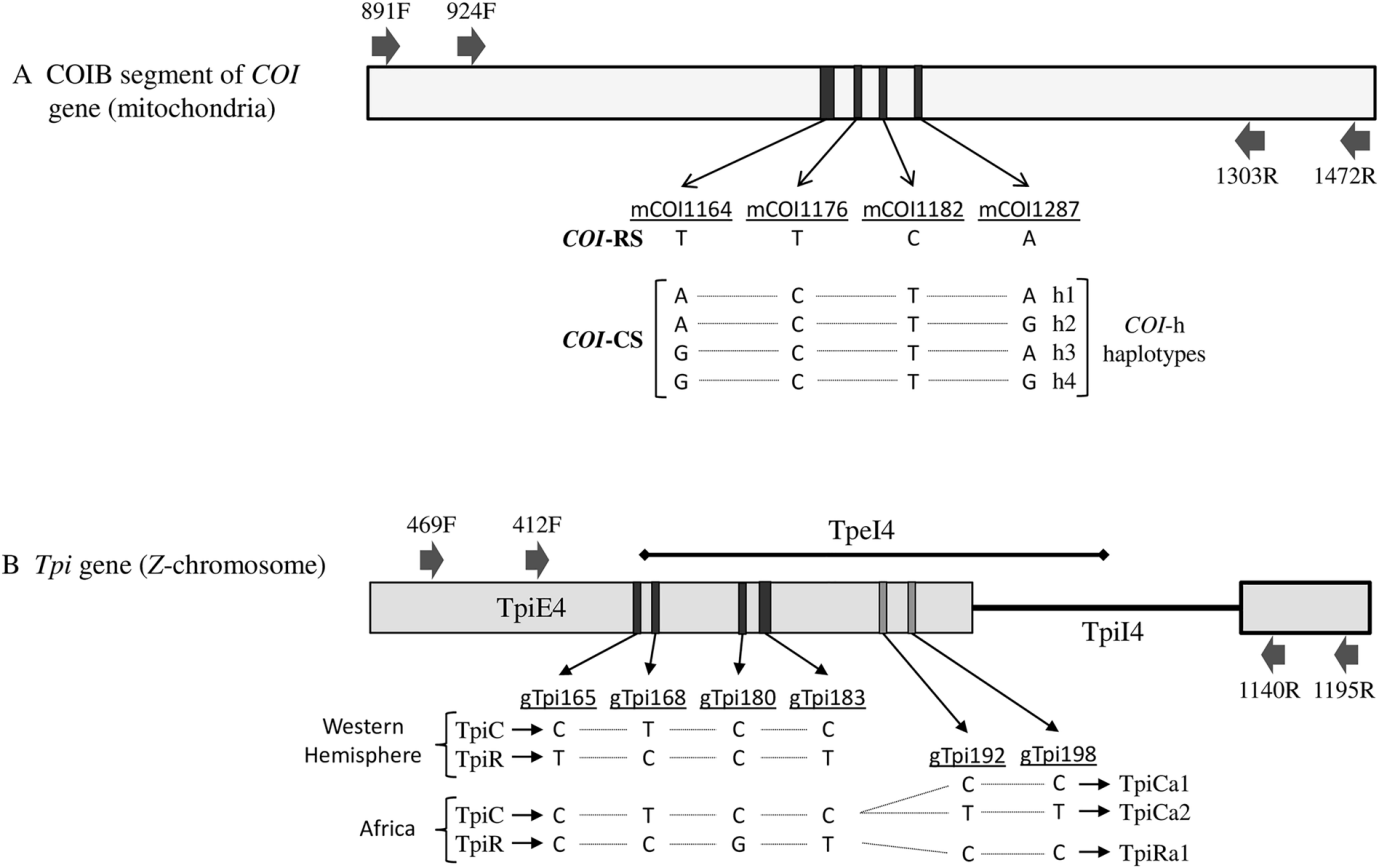
Diagrams of the relevant *COI* and *Tpi* gene segments, with descriptions of polymorphisms used to identify different haplotypes. Vertical lines in gene segments indicated site of single-base polymorphism with the thicker band indicating polymorphism diagnostic of strain identity. (**A**) COIB polymorphisms and the haplotypes observed in African FAW. Sites mCOI1164D and mCOI1287R identify strain and the h-haplotypes (h1-4). Sites mCOI1176 and mCOI1182 are also strain-specific in Western Hemisphere populations. (**B**) Diagram of the *Tpi* sequence used to PCR amplify the TpiE4 exon segment. Site gTpi183 is diagnostic of Tpi-based strain identity in all locations. Sites gTpi165 and gTpi168 are also strain-specific in the Western Hemisphere. Sites gTpi168 and gTpi180 are also strain-specific in Africa. Sites gTpi192 and gTpi198 are polymorphic but not strain-specific in all locations. Block arrows indicate location of primers used for PCR amplification and DNA sequencing.

The specimens were of variable quality and in many cases a single PCR amplification did not produce sufficient product. In these cases, a nested PCR protocol was performed. For COIB analysis the first PCR amplification was performed with the primer pair *CO1-891F* and *CO1-1472R*. One microliter of this first reaction was amplified using primers *CO1-924F* (5′-TTATTGCTGTACCAACAGG-3′) and *CO1-1303R* (5′-CAGGATARTCAGAATATCGACG-3′). For the *Tpi* marker, the first amplification was performed using primers *Tpi469F* (5′-AAGGACATCGGAGCCAACTG-3′) and *Tpi1195R* (5′-AGTCACTGACCCACCATACTG-3′). One microliter of the first reaction was then amplified using primers *Tpi412F* and *Tpi1140R*. Relative locations of the primers are described in Fig. 2.

### Determination of strain-identity using *COI* and *Tpi*

To facilitate the analysis of large numbers of samples, strain identity was initially defined by a single site in *COI* or *Tpi* as previously described^26^, with the identification confirmed by comparisons with nearby strain-specific SNPs (Fig. 2A,B). Designation of the *COI* and *Tpi* gene sites are preceded by an "m" (mitochondria) or "g" (genomic), respectively. This is followed by the gene name, number of base pairs from the predicted translational start site for *COI*, or the 5′ start of the exon for *Tpi* and, when relevant, the nucleotide observed. The COIB segment lies in the 3′ half of the *COI* gene. *COI*-based strain identity was determined by mCOI1164, with an A or G signifying C-strain and a T indicating R-strain (Fig. 2A). Sites mCOI1176 and mCOI1182 are similarly strain specific. Strain identity by *Tpi* is defined by gTpi183 found in the fourth exon (TpiE4) of the predicted *Tpi* coding region (Fig. 2B). Site gTpi168 shows the same strain-specific pattern as gTpi183. Two other sites display hemispheric differences in their association with strain identity due to differences in the R-strain. The nucleotide T is found at gTpi165 (gTpi165T) that correlates with gTpi168C and gTpi183T in Western Hemisphere FAW populations but not in FAW surveyed in Africa^26,28^. Site gTpi180 shows the reciprocal pattern with gTpi180G correlating with R-strain associated gTpi183T in Africa FAW but not with those in the Western Hemisphere.

A complication of using the *Tpi* marker is that male FAW (*Z/Z*) carry two copies of the *Tpi* gene and so can be heterozygous (TpiR/TpiC) for the strain markers. These heterozygous specimens (TpiH) are identified by DNA sequencing chromatographs that show overlapping C and T curves at gTpi183, as well as overlapping C/G and C/T curves at gTpi180 and gTpi168, respectively. The number of TpiH specimens were incorporated into the calculations of TpiC and TpiR frequencies. In collections from pheromone traps, all specimens are male and therefore carry two copies of the *Tpi* gene, with TpiH carrying one copy each of TpiC and TpiR. In these collections the number of the TpiC haplotype was calculated by 2(number of TpiC specimens) + (number of TpiH specimens) and the TpiR haplotype by 2(TpiR) + (TpiH). In the larval collections gender was typically not identified. In this case a 1:1 sex ratio was assumed with half the collection considered male. In these collections the number of *Tpi* haplotypes was calculated as 1.5 (TpiC) + (TpiH) or 1.5 (TpiR) + (TpiH) for TpiC and TpiR frequencies, respectively.

### DNA sequence and statistical analysis

DNA sequence alignments and comparisons were performed using programs available on the Geneious 10.0.7 software (Biomatters, Auckland, New Zealand). Basic mathematical calculations and generation of graphs were done using Excel and PowerPoint (Microsoft, Redmond, WA). Other statistical analyses including *t*-tests were performed using GraphPad Prism version 9.1.0 for Mac (GraphPad Software, La Jolla, CA, USA). ANOVA calculations were combined with Tukey multiple comparisons testing to make pair-wise comparisons.

## Results

### Regional distribution of genetic markers

Two earlier surveys found evidence for FAW population structure with respect to the frequencies of the mitochondrial *COI* strain markers and a *Tpi* exon variant designated TpiCa2 (Fig. 2B)^16,26^. FAW collected from the western African nations of Togo and São Tomé and Príncipe (TogS&P) had significantly higher frequencies of the *COI*-CS and TpiCa2 haplotypes than those from four more eastern African nations (Table 2a,b)^16^, and this difference was still observed with the inclusion of data from five additional eastern locations (Table 2c,d)^26^.

**Table 2:** Statistical comparisons between selected FAW collections using two-tailed t-test (TogS&P = Togo and São Tomé and Príncipe group).

	Comparison (letters from Table 1)	*t*-statistic	*df*	*P*-value
a	*COI*-CS: TogS&P (dh)^1^ vs East (mpqrt)^a^	4.55	5	0.0061
b	TpiCa2: TogS&P (dh)^1^ vs East (mpqrt)^a^	4.14	5	0.0090
c	*COI*-CS: TogS&P (dh)^2^ vs East (cjkmpqrtuw)^b^	6.81	11	< 0.0001
d	TpiCa2: TogS&P (dh)^2^ vs East (cjkmpqrtuw)^b^	3.07	11	0.0107
e	*COI*-CS: West (a-m) vs East (n-w)	3.30	27	0.0028
f	TpiCa2: TogS&P (dh) vs East (n-w)	2.25	14	0.0411
g	TpiCa2: West (a-m) vs East (n-w)	1.70	29	0.1004
h	TpiC: West (a-m) vs East (n-w)	1.37	27	0.1816
i	West (a-m):* COI*-CS TpiC vs *COI*-RS TpiC	1.40	34	0.1721
j	East (n-w):* COI*-CS TpiC vs *COI*-RS TpiC	7.91	20	< 0.0001
k	*COI*-CS TpiC: West (a-m) vs East (n-w)	2.96	27	0.0063
l	*COI*-RS TpiC: West (a-m) vs East (n-w)	4.38	27	0.0002
m	TpiH: West (a-m) vs East (n-w)	1.10	27	0.2803

^a^Only collections from 2016 to 2017^16^.

^b^Only collections from 2016 to 2017^26^.

**Figure 3:**
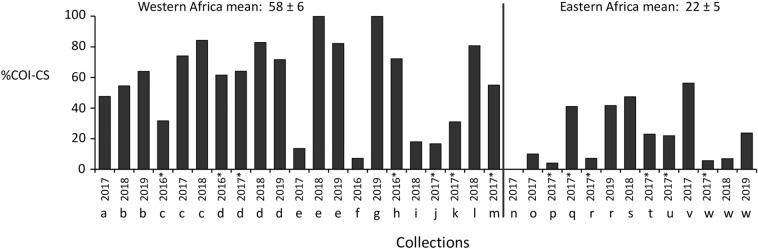
Bar graph describing *COI* haplotype frequencies in western and eastern Africa. Collections are as described in Table 1. The mean ± the Standard Error of the Mean (SEM) for different regions is presented above the graph. Asterisks indicate data from earlier studies.

In the current study the survey was expanded by 24 previously uncharacterized collections from 2016 to 2019 representing an additional 11 nations. This total of 34 collections were subdivided into two groups, western Africa (21 collections) and eastern Africa (13 collections, Fig. 3), based on the distribution of major agricultural areas that tend to concentrate in regions near the Atlantic and Indian oceans (Fig. 1). A statistically significant difference was observed in the *COI*-CS frequency between the groups (Table 2e). On average a majority of 58% of the FAW tested from the western African collections were *COI*-CS compared to a mean of 22% in the eastern group (Fig. 1). Multi-year data were available for six countries, Senegal (b), Ghana (c), Togo (d), Benin (e), Tanzania(r), and South Africa (w, Fig. 3). The *COI*-CS frequency was generally consistent over time at each location with two major exceptions. In Ghana 2016 and Benin 2017, *COI*-RS was the majority haplotype while *COI*-CS predominated in the subsequent 2 years.

**Figure 4:**
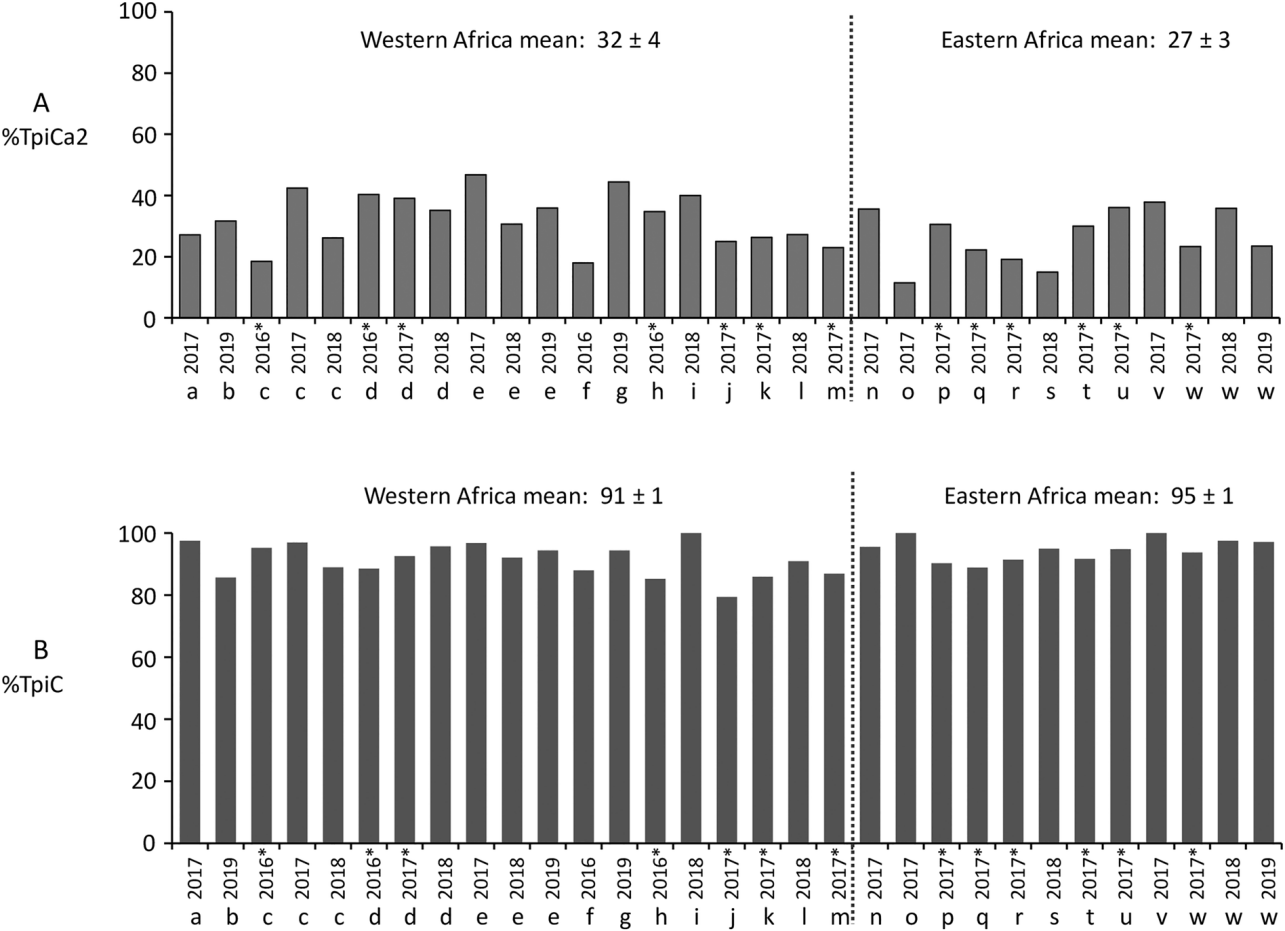
Bar graphs indicating frequency of *Tpi* haplotypes in western and eastern Africa. Collections are as described in Table 1. The mean ± SEM for different regions is presented above the graph. Asterisks indicate data from earlier studies. (**A**) Frequency of the TpiCa2 haplotype. (**B**) Frequency of the TpiC haplotype diagnostic of the C-strain.

With respect to the TpiCa2 haplotype, the previously observed significant differences between TogS&P versus other African collections was preserved even with the addition of new eastern collections (Table 2f). When the TogS&P pool was expanded to include other collections from the western region, the mean TpiCa2 frequency was still higher than in the east, 32% compared to 27% (Fig. 4A). However, this difference was no longer statistically significant (Table 2g).

### Distribution of FAW strains and hybrids

All the African specimens tested were collected from C-strain preferred host plants or habitats (corn or sorghum), leading to the expectation that the C-strain should be the majority in all collections. However, the *COI*-CS data suggests a differential distribution of the strains by geography, with the C-strain the majority in western African countries and the R-strain predominating in the more eastern and southern locations. This possibility was tested by examining the distribution of the *Tpi* strain markers in Africa. The TpiC haplotype, which is diagnostic of the C-strain, predominated in all locations with an overall mean frequency of 87% and a range of 74%-100% (Fig. 4B), with no significant difference in the mean TpiC frequency observed between western (91%) and eastern (95%) African FAW populations (Table 2h). Therefore, unlike the *COI* markers, the observed TpiC haplotype frequencies agree with expectations from host plants.

**Figure 5:**
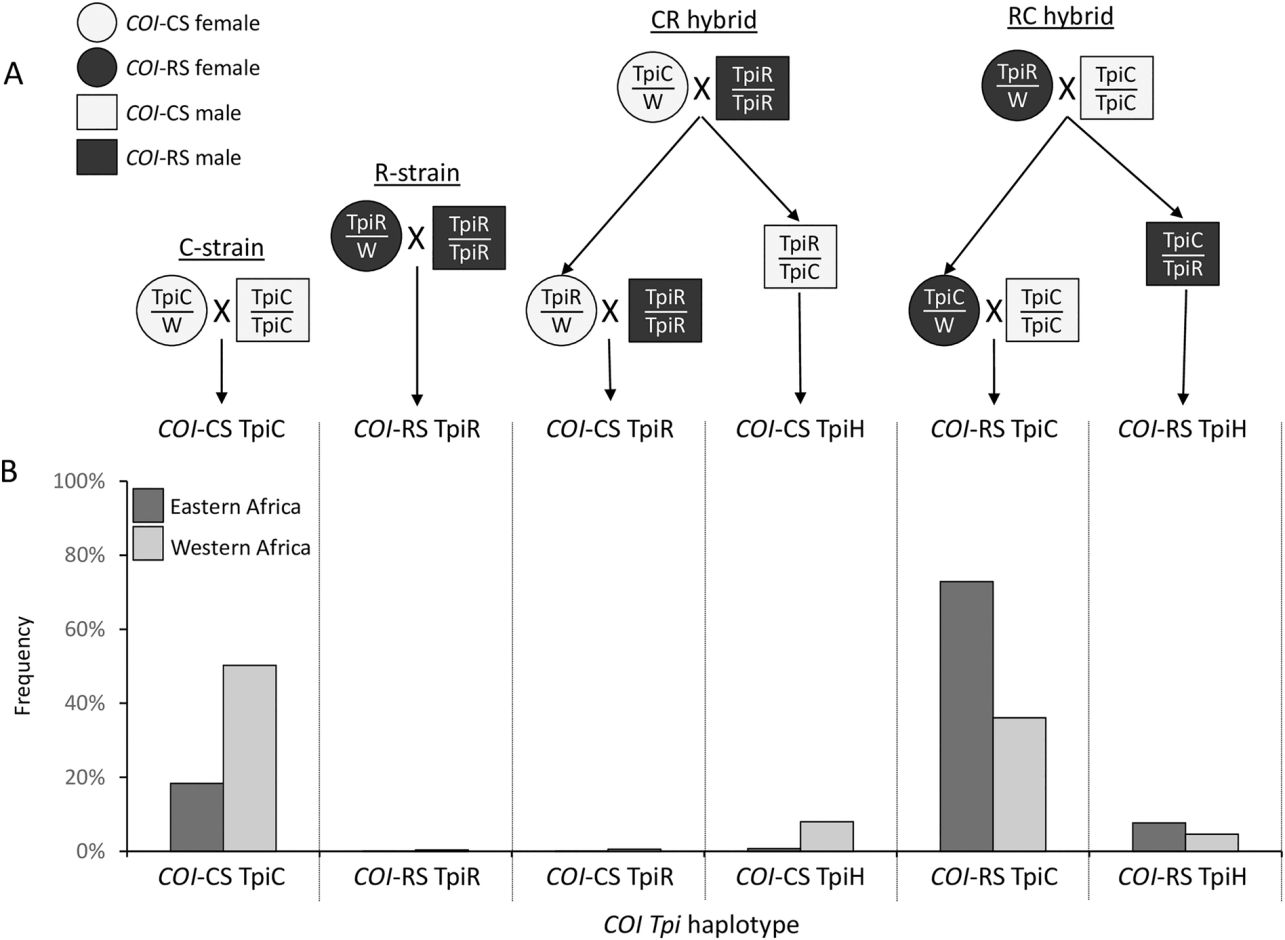
Description of origins and frequencies of the different observed *COI Tpi* haplotype configurations. (**A**) Describes crosses that can produce different *COI Tpi* combinations. The *COI* haplotype is maternally inherited and indicated by either a circle (female) or square (male), with shading differentiating *COI*-CS from *COI*-RS. The *Z*-linked *Tpi* gene is present in one copy in females (*Z/W*) and two copies in males (*Z/Z*) and undergoes sex-linked segregation. “CR hybrid” indicates cross between a C-strain female and R-strain male, while “RC hybrid” defines the reciprocal mating. (**B**) Describes the frequencies of the different *COI Tpi* configurations in western and eastern Africa.

A subset of the specimens was analyzed with both *COI* and *Tpi* strain markers. Figure 5A describes examples of the crosses both within and between strains that can explain the observed *COI* and *Tpi* haplotype combinations. The *COI* marker is mitochondrial and so is inherited maternally, while the *Tpi* gene is on the Z-chromosome and displays sex-linked segregation. Figure 5B describes the results from 1197 specimens (404 from eastern Africa, 793 from western Africa) where both *COI* and *Tpi* sequence data were available. Two genotypes predominated in Africa. The *COI*-CS TpiC configuration that is concordant for both C-strain markers is the most common configuration in western Africa with a mean frequency of 50% that is not significantly different from the 36% frequency of the discordant *COI*-RS TpiC group (Fig. 5B, Table 2i). In contrast, the discordant *COI*-RS TpiC configuration predominates in eastern Africa at a frequency (73%) significantly higher than the *COI*-CS TpiC haplotype (18%, Table 2j). Overall, *COI*-CS TpiC frequencies in the western Africa group was significantly higher than in the eastern group (Table 2k) and, conversely, *COI*-RS TpiC was significantly more frequent in the east than west (Table 2l).

Consistent with previous studies the frequency of TpiR remains very low^14,27^. From a total of 1722 specimens tested only 18 were TpiR. These were found in Benin (3 specimens), the Democratic Republic of Congo (2), Ghana (2), South Africa (2), Senegal (2), and Togo (6), representing less than 5% of the specimens from each location. All 18 TpiR were identical in sequence for the 237 bp TpeI4 segment previously described and deposited into GenBank (MH729873)^14^. Another 211 African specimens produced sequence chromatographs with overlapping curves at certain polymorphic sites that are indicative of a TpiC/TpiR heterozygote (TpiH). The mean TpiH frequency was higher in western Africa (13%) than eastern (8%), but the difference was not statistically significant (Table 2m). The TpiH specimens were found in all countries except for Ethiopia, Gabon, and Zimbabwe (where sample sizes were relatively low, < 15). This indicates that TpiR is broadly distributed in Africa but is rare and usually found in combination with TpiC as a likely hybrid.

### Evidence of a new FAW introduction into western Africa

**Figure 6:**
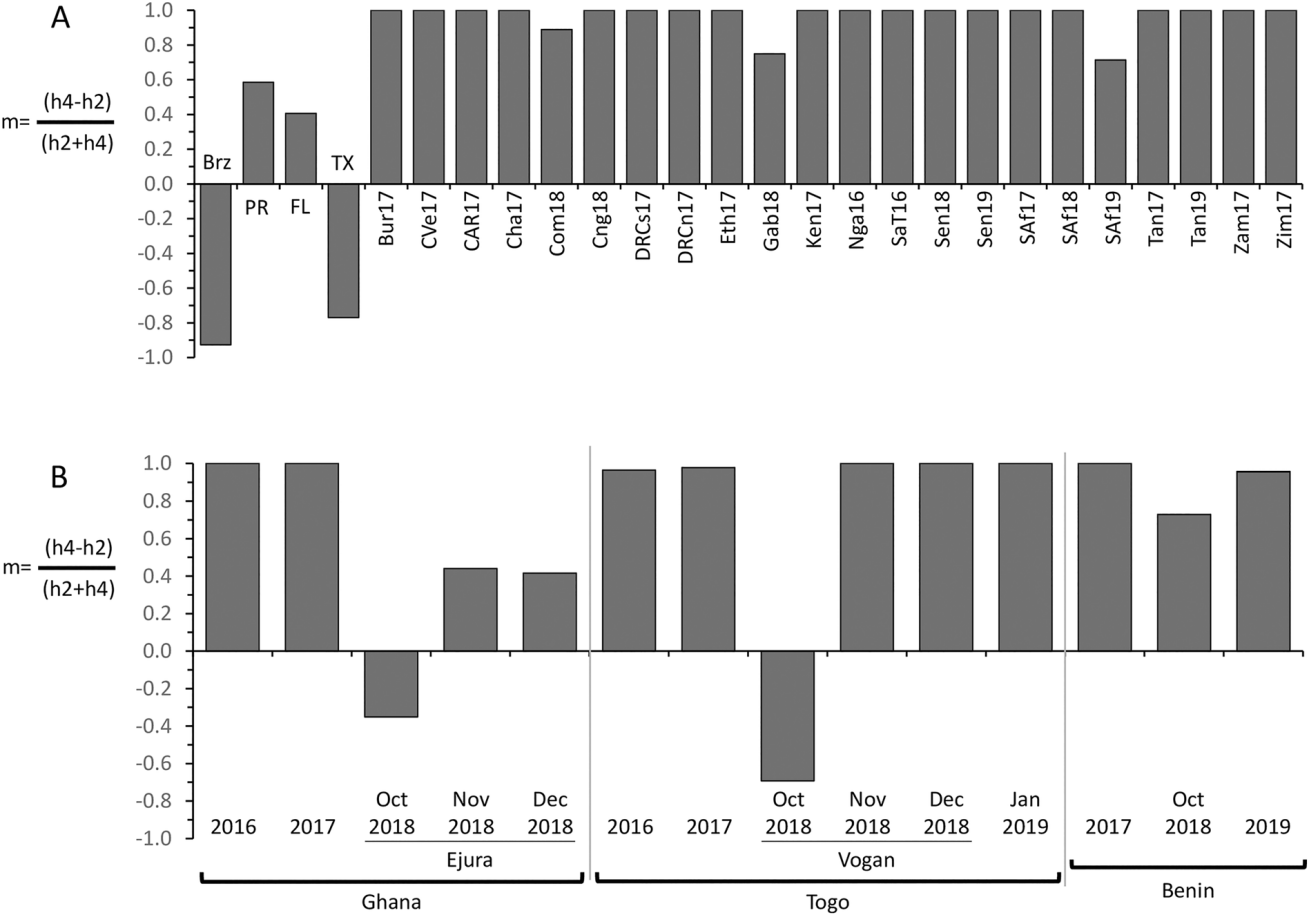
Bar graphs describing the m-values calculated for different collections as described in Table 1. (**A**) compares the m-values for various sites in Africa with those observed with FAW from Brazil (Brz), Puerto Rico (PR), Florida (FL), and Texas (Tx). (**B**) describes the m-values for collections from Ghana, Togo, and Benin.

The COIB segment contains SNPs that subdivide the *COI*-CS haplotype into four variants, *COI*-h1-4 (Fig. 2A), that are found throughout the Western Hemisphere but consistently differ in their relative proportions in a manner that identifies two geographically distinct groups^32,33^. Specifically, the *COI*-h4 variant is the predominant form in populations that winter in Florida and most of the Caribbean while *COI*-h2 is the majority in South America and populations that winter in Texas and Mexico^18,21,32,34^. Using the metric m = (h4 − h2)/(h2 + h4) we consistently find that populations from Florida and Puerto Rico give positive values while those from Texas and South America are negative (Fig. 6A)^13,32^. Our past surveys of FAW from Africa found a predominance of *COI*-h4 in all tested locations and this was generally confirmed by the additional collections in this study. We found that all the collections outside of Ghana and Togo that span most of the African continent were strongly positive, with a mean of + 0.97 (Fig. 6A).

Similar results were obtained for collections from multiple corn sites in Ghana in 2016 and 2017 where the *COI*-h2 haplotype was not found, resulting in an m-value of + 1.0 (Fig. 6B). In contrast, October 2018 collections from corn fields in Ejura, Ghana showed a preponderance of the *COI*-h2 haplotype (25 *COI*-h2 compared to 12 *COI*-h4) giving an m-value of − 0.35. Ejura is within 50 km of sites sampled in 2016 and 2017 and so these specimens are expected to be part of the same interbreeding population. Collections from Ejura in subsequent months show a return to positive m-values, though at levels below that observed in 2016–2017 (Fig. 6B).

A similar pattern was observed in collections from adjacent country of Togo. FAW from multiple locations in Togo (including the inland Vogan zone) in 2016 and from the more coastal Lomé area in 2017 gave positive m-values of + 0.96 and + 0.98, respectively, with only four specimens expressing the *COI*-h2 haplotype out of 513 tested (Fig. 6B). But in October 2018, collections from corn in the Vogan zone had an m-value of − 0.69, with 11 of the 18 specimens being h2 and four specimens *COI*-h1, a haplotype that has so far not been found elsewhere in Africa and is a minor haplotype in the Western Hemisphere. However, subsequent collections from the Vogan zone from November and December 2018 showed highly positive m-values with no *COI*-h1 or *COI*-h2 haplotypes detected. Contemporaneous collections from pheromone traps in the neighboring country of Benin showed only positive m-values ranging from + 0.73 to + 1.00 but with the lowest score occurring in October 2018 (Fig. 6B).

## Discussion

The results of this updated survey of the African FAW are in general agreement with past studies and lead to three observations with potentially important implications concerning the entry and migration of FAW within the continent. The first is that previously observed regional differences in the distribution of the *COI*-CS haplotype persists even after the inclusion of additional sites and later collections. The second is that the African FAW population is dominated by the C-strain and a lineage that appears to be derived from an interstrain hybridization event, with the R-strain continuing to be mostly absent. The third is evidence of a new incursion of FAW into western Africa that appears to be from a different source than the previous introduction. The potential ramifications of these observations are discussed below.

A map of the distribution of farmland in Africa shows concentrations along the western and eastern coastal nations separated by large covers of natural forest with relatively little agricultural activity (Fig. 1). This pattern suggests limited availability of preferred host plants for FAW in the central area that could restrict west–east movements of large populations by natural migration, thereby explaining the persistence of the *COI*-CS asymmetry first observed in collections from 2016 to 2017^16^ and still detected in this study. However, such limitations in migration run counter to the prevailing invasion scenario of a recent introduction of FAW into western Africa followed by the rapid migration to the rest of sub-Saharan Africa in the next two years. While the observation of a small number of identical *COI* and *Tpi* haplotypes throughout Africa strongly suggests a single, common source population^16^, regular and substantial migration at the continental scale should result in the homogenization of haplotype frequencies as well. Therefore, the persistence of the east–west difference in *COI*-CS frequency indicates that transcontinental movements of large numbers of FAW by natural migration is limited and suggests that smaller scale movements through trade probably played a significant role in the initial spread of this pest across Africa.

Other moth species also show evidence of a divide between populations in western and eastern Africa. In particular, the noctuid moth *Busseola fusca* (Fuller) and the pyralid *Eldana saccharina* (Walker) both show clades defined by mitochondrial haplotypes that are geographically separated in a manner sharing broad similarity to that displayed by FAW^35–37^. Both *B. fusca* and *E. saccharina* are native to Africa with the observed segregation attributed to geological and climatic events dating back to the Miocene and Pleistocene eras. The persistence of these phylogeographic patterns to the present day suggests the existence of significant physical barriers to natural migration on the African continent that impede homogenization and would be expected to impact the distribution and mixing of FAW populations.

The differential distribution of the *COI*-CS and *COI*-RS haplotypes in Africa is particularly interesting because these are commonly used markers for identifying the FAW host strains in Western Hemisphere populations. The strains differ in their association with plant hosts in the field, with the C-strain preferentially found in corn, sorghum, and cotton while the R-strain preferring turf and pasture grasses, alfalfa, millet, and rice^20,21,38^. The determination of what strains are present is critical to assessments of what crops are at risk of significant FAW infestations. However, such assessments are complicated by the fact that the strains are morphologically indistinguishable and so can only be identified by a small number of molecular markers that have so far been limited to genetic elements that map to mitochondria (such as *COI*^17^) or the sex chromosomes (i.e., *Tpi*^18^, *FR*^39^, *esterase*^19^). The association of the *COI* and *Tpi* strain markers with FAW collected from different plant host species has been consistently observed in surveys from both Americas, indicating that the strains are broadly distributed and a general characteristic of the species^22,23,40^. However, this correspondence is not absolute. For example, typically about 20% of FAW collected from corn hosts in the Americas display R-strain markers, and there are multiple FAW collections from corn or rice host plants where a majority will display the opposing strain markers^21,24,41^. These observations suggest that the association between FAW strain and plant host is more of a preference than a requirement, consistent with laboratory feeding studies indicating that both strains can successfully develop on the same set of plant hosts^42,43^. In addition, while reproductive barriers between the strains have been observed, they are incomplete, with successful hybridization between strains demonstrated in the laboratory and evidence of significant hybrid frequency found in field populations^19,44–47^. The hybrids appear to differ from the parental strains with respect to mating behavior and reproductive compatibility^44–46^, but the impact on plant host preference remains uncertain.

Because gene flow between strains is directly dependent on the formation of interstrain hybrids, we expect that the amount of strain divergence at any location will be impacted by whether and to what degree differential plant host preferences deter mating between strains. If this factor is significant, then divergence should tend to increase in locations where the primary hosts for each strain are abundant and separated, as under these conditions the strains can remain segregated. In contrast, in habitats with less host variety or abundance the two strains are more likely to overlap out of necessity, increasing the likelihood of cross hybridization. Given these considerations, it is likely that FAW displays a complex population structure made up of the C-strain, the R-strain, and inter-strain hybrids, where the proportion of each group and the frequency of mating between and within groups will depend upon the types and distributions of local plant hosts. If correct, then the degree of divergence between the two strains as measured by genetic differentiation could vary significantly by location. This scenario could explain the contradictory results from recent studies where differences between the strains at the whole genome level were detected in some comparisons^48^, but not in others^49^.

The situation in Africa differs from the Americas in that while both *COI*-CS and *COI*-RS are detected at high frequency, the TpiR haplotype is very rare in Africa, present in less than 1% of all specimens even in collections from habitats dominated by R-strain preferred plant hosts^27^. The Africa population is dominated by two *COI Tpi* configurations, *COI*-CS TpiC present in 41% of specimens and *COI*-RS TpiC at 47%, with the former preferentially found in the western Africa grouping of collection sites and the latter in eastern Africa (Fig. 3). The *COI*-CS TpiC configuration is normally representative of the C-strain and is the predominant haplotype found in specimens from cornfields in the Western Hemisphere^16,21,22^. The *COI*-RS TpiC is a configuration predicted to arise from an interstrain cross between a R-strain female and C-strain male with the daughters then back crossed to C-strain males (*COI*-RS TpiC hybrid, Fig. 5A). This configuration is found at variable frequencies in the Western Hemisphere, with the highest frequencies associated with C-strain preferred host plants^18,24^. These observations suggest that the *COI*-RS TpiC population is behaving as the C-strain with respect to plant host use and this seems to be the case in Africa as reports of agricultural damage by FAW has consistently been limited to C-strain plants like corn and sorghum^7,27^.

Given these results, we believe that the *COI*-RS TpiC population should be considered part of the C-strain group despite its derivation from an interstrain hybrid mating. Justification for this assumption comes from consideration of the effectiveness of *Z*-linked markers such as *Tpi* to distinguish strains, which can best be explained if the primary determinants for strain identity are also on the *Z*-chromosome and therefore physically linked to the *Tpi* gene. This proposition is consistent with observations that genetic differences between lepidopteran species are disproportionately sex-linked^50^. Based on this reasoning, it is likely that strain identity is defined primarily, if not solely, by the *Z*-chromosome, and we note that the TpiC marker indicates that the Z-chromosome of both *COI*-CS TpiC and *COI*-RS TpiC is of the C-strain. If these assumptions are correct, then we anticipate no significant differences in the behaviors of the *COI*-CS TpiC and *COI*-RS TpiC groups and suggest that their differential distribution across western and eastern Africa is probably due to chance.

The evidence of a second FAW introduction into Africa comes from what appears to be an influx of the TX-type *COI*-h2 haplotype in 2018 into Ghana, Togo, and perhaps Benin (Fig. 6B). In collections from October to December 2018, the *COI*-h2 haplotype made up 32% (51/162) of specimens from Ejura, Ghana, with a peak in October where it was the majority form, 58% (25/43). *COI*-h2 was only detected in October in Vogan, Togo, where it made up 61% (11/18) of specimens, while Benin showed 14% (5/37) during the same month. In contrast, in all other African locations, including collections from Togo, Ghana, and Benin from other years, the pooled *COI*-h2 frequency was only 0.5% (9/1501) with a range from 0.00 to 0.02%. Once introduced, the *COI*-h2 haplotype would be expected to disperse into the much larger *COI*-h4 population and become increasingly difficult to detect. This appears to be what occurred as the *COI*-h4 haplotype again predominated in Vogan, Togo and to a lesser extent in Ejura, Ghana after October 2018 (Fig. 5B). This persistence of lower m-values in Ejura in November and December 2018 is of potential interest as it could indicate that the *COI*-h2 haplotype is becoming established in the area, which might happen if the dispersion and mixing of populations in Ejura is less than occurs at the Vogan site. Continued monitoring of Ejura FAW is needed to determine if this represents a long-term shift in *COI*-h haplotype proportions.

If this incidence of *COI*-h2 does represent a new incursion it appears to be from a different source than that of the original introduction that gave rise to the predominantly FL-type *COI*-h4 composition of the Africa population. The possibility of a second incursion of Western Hemisphere FAW into western Africa is troubling as it suggests that the conditions that allowed for the first introduction are still in place despite efforts to improve monitoring and food security. This means that FAW subpopulations of concern thought to be currently rare or absent in Africa could be introduced at any time. This includes the R-strain, which would put important crops like rice, millet, and forage grasses at risk, and FAW lines capable of developing on corn expressing the Cry1F Bt-product in Puerto Rico^51^.

How FAW reaches Africa from the Western Hemisphere is an important issue that remains unclear, with trade the most likely mechanism. Using the European Union (EU) as an example, assessments based on FAW intercept frequencies at EU ports of entry suggest that as many as a million FAW larvae could enter the EU annually^52,53^. A related study in Australia found evidence that sea cargo containers are routinely exposed to and often unintentionally carry economically important insects, potentially serving as a conduit for the spread of invasive pests^54^. These observations are consistent with our findings that the threat of new introductions of FAW into Africa is significant.

In summary, the results for this genetic survey of FAW in Africa demonstrate the value of continued surveillance of pest populations at the continental level. FAW is in the process of becoming established in Africa with the distribution of permanent populations and pattern of regional migrations still to be determined. Identification of genetic structure as found for the *COI*-CS haplotype can define the magnitude and limits of natural migration. Evidence of a second incursion of FAW, most likely from the Western Hemisphere, indicate that continued introductions are plausible, which could rapidly alter the composition of the Africa population with respect to pesticide resistance and host range.
